# Computer algorithm can match physicians’ decisions about blood transfusions

**DOI:** 10.1186/s12967-019-2085-y

**Published:** 2019-10-10

**Authors:** Yuanyuan Yao, Jenny Cifuentes, Bin Zheng, Min Yan

**Affiliations:** 1 Department of Anesthesiology, The Second Affiliated Hospital of Zhejiang University, Hangzhou, China; 2 Program of Electrical Engineering, Universidad De La Salle, Bogotá, Colombia; 3 Department of Surgery, University of Alberta, Edmonton, Canada; 4 Department of Anesthesiology, the Second Affiliated Hospital, School of Medicine, Zhejiang University, Hangzhou, 310009 China

**Keywords:** Artificial intelligence, Neural networks (computer), Computer algorithm, Blood transfusion, Patient safety, Surgery

## Abstract

**Background:**

Checking appropriateness of blood transfusion for quality assurance required enormous usage of time and human resources from the healthcare system. We report here a new machine learning algorithm for checking blood transfusion quality.

**Materials and methods:**

The multilayer perceptron neural network (MLPNN) was designed to learn an expert’s judgement from 4946 clinical cases. The accuracy in predicting the blood transfusion was then reported.

**Results:**

We achieved a 96.8% overall accuracy rate, with a 99% match rate to the experts’ judgement on those appropriate cases and 90.9% on the inappropriate cases.

**Conclusions:**

Machine learning algorithm can accurately match to human judgement by feeding in pre-surgical information and key laboratory variables.

## Background

Blood transfusion is a critical step for saving lives in many clinical situations. However, blood, as a valuable resource, is often scarce in hospitals around the world [1–6]. While many people and resources are dedicated to increasing blood supply by promoting blood donation and storage safety, [7, 8] physicians’ behaviors in making the decision for blood transfusion should also be investigated.

For patients undergoing surgical procedures, the decision for blood transfusion should primarily be made on the basis of the volume of blood loss associated with the surgery and adjusted by pre-surgery factors, such as the American Society of Anesthesiologists (ASA) score, age, history of anemia, and accompanying chronic diseases of the cardiopulmonary and renal systems [9, 10].

Worldwide, compliance with the WHO guideline is problematic [11, 12]. For various reasons, physicians and surgeons often over-prescribe blood transfusions in clinical practice [13]. In our recent survey of patients undergoing surgical care in 9 hospitals in China, the rate of inappropriate ordering of blood transfusion was as high as 37% in 2007 [14]. Therefore, there is a need to constantly survey physician’s prescription of blood transfusion and to check its appropriateness.

Checking appropriateness of blood transfusion is tedious work. To enhance the effectiveness of data monitoring, we report here our practice of creating a computer algorithm to examine the appropriateness of blood transfusion decisions using machine learning strategy.

Machine learning is not a new concept, but it has made significant achievements and gained public attention after a few significant breakthroughs in recent years [15]. In the core of machine learning sits the artificial neural network (ANN), which is a mathematical analogue for describing how multiple factors interact with each other before a decision is made.

When applying the ANN model for medical research we need to carefully select input variables, and decide the training size and structure (Walezak 2005). In this paper, we used Restricted Boltzman Machines (RBM) to help us in finding the optimal parameters feeding to the ANN model to improve the accuracy of prediction. By applying machine learning algorithm, we expect to find a way to quickly check the appropriateness of blood transfusion on a large volume of cases. We expect the outcome from computer reports can be similar to human judgement.

## Materials and methods

### Data: blood transfusion cases

The blood transfusion dataset was acquired from the Department of Anesthesiology in the Second Affiliated Hospital of Zhejiang University in China. The dataset included 15,000 surgical cases collected from 9 hospitals in Zhejiang, China in 2007 and 2011. The study protocol was reviewed and approved by the Ethics Review Committee at the Second Affiliated Hospital of Zhejiang University. An initial report of this work was published in 2018 [14].

After anonymization of patient identification, a spreadsheet with a total of 4946 data entries (i.e., patients who accepted intra-operative blood transfusion) was compiled. The dataset was received by scientists at the Surgical Simulation Research Lab at the University of Alberta in order to develop a custom-designed computer algorithm. All of the transfusion cases were adult hospitalized patients; they underwent a variety of surgical procedures in 2007 and 2011.

### Algorithm: multilayer perceptron neural network

Details of running the Restricted Boltzman Machines (RBM) method for selecting the optimal parameter is reported in another technological paper. In brief, a list of parameters were elected for each case (Table 1).

**Table 1:** The information of collected variables

Demographic information
Hospital
Preoperative anemia
Age
Weight
ASA grade (I–V)
Type of admission
Comorbidities
Hypertension
Cardiaovascular problems
Chronic obstractive pulmonary disease
Diabetes
Operation information
Type of surgical procedure
Duration of surgery
Volume of blood lost in surgery
Hemogrobin (Hb) level before transfusion

The predicting variable is the appropriateness of the blood transfusion. In each case, the appropriateness was judged by a group of experienced anesthesiologists and based on the WHO Handbook and *Guidelines for Clinical Use of Blood* issued by the Ministry of Health of the People’s Republic of China [9, 16].

A multilayer perceptron neural network (MLPNN) with 50 hidden neurons was created in the computer algorithm. The 4946 data entries were inputted to the model using the script in Python. The goal for machine learning was to let the computer match an expert’s judgement on the appropriateness of each blood transfusion. In this way, 2 output neurons were implemented, representing the “need” and “not need” of blood transfusion.

By feeding the computer algorithm with sufficient data, the computer will gradually find the best bias and weight values for each intra-neural interaction until the output from the mathematical model can match human judgement. After training finishes, the accuracy of the resulting MLPNN model’s parameters are compared to the human (expert) judgement.

**Fig. 1:**
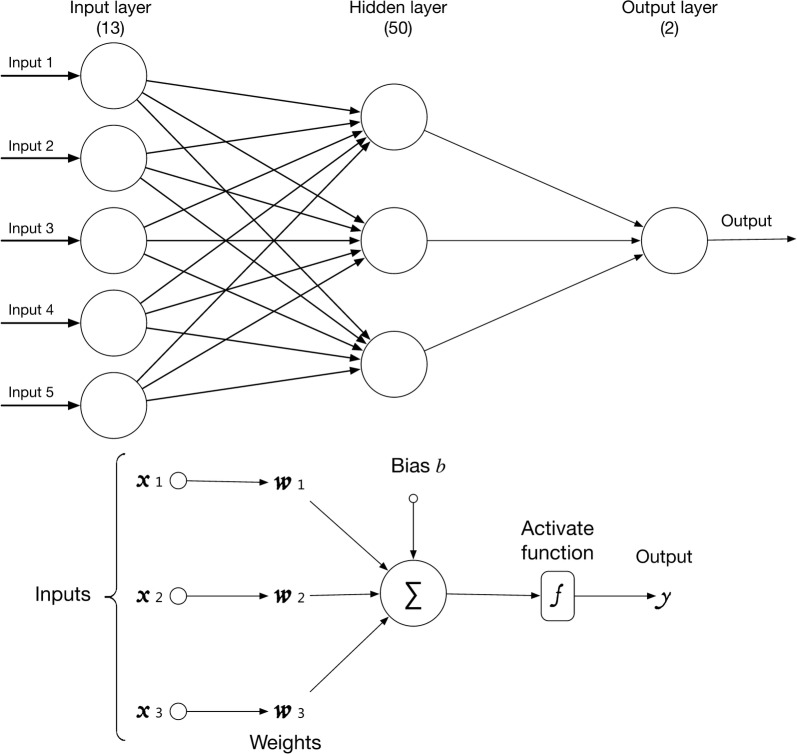
Multilayer perceptron neural network structure

Illustration of the neural network used in this project is highlighted in Fig. 1. Details of the computer script for screening the appropriateness of blood transfusions can be found in the Additional file 1: Appendix S1.

## Results

Of the total 4946 patients who received intra-operative blood transfusions, expert anesthesiologists rated 3604 cases as appropriate and 1342 as inappropriate on the basis of WHO guidelines.

Our algorithm was trained by these data, and as such, achieved a 96.8% accuracy rate in matching human judgement: 99% of the computer’s decisions matched the experts on the appropriate cases and 90.9% matched on the inappropriate cases. There was a 1% false negative rate for the appropriate case (i.e., the expert considered a blood transfusion appropriate but the computer did not agree). There was a 9.1% false positive rate for the inappropriate cases (i.e., the human expert considered the blood transfusion as not needed but the computer gave permission for a transfusion). Table 2 lists the classification outcomes.

**Table 2:** Classification results

	Physician judgement		General classification
	Need transfusion	No need transfusion	
Computer report			
Yes	True positive 3569/3604 (99.0%)	False negative 122/1342 (9.1%)	
No	False positive 35/3604 (1.0%)	True negative 1220/1342 (90.9%)	
			96.8% (3569 + 1220)/4946 × 100%

A total of 4946 entries, with 3604 about “need”, and 1342 for “no need”

## Discussion

We would like to first discuss the discrepancy between physician and computer’s judgement on blood transfusion; specifically 9.1% false negative rate requires a good explaination. A quick response to this results is that the discrepancy may also come from human bias. After years’ education for reducing unnecessary blood transfusion, our experts may adopte a habit of being criticised on transfusion cases. We need to be awared of this phenoment when we perform similar research in future involving with different group of experts and under different health organizations.

By checking 122 cases where computer gave a permission of transfusion but physicians denied (false negative), a majority of those cases were accompanied with extra health problems or with a more critical condision such as with a higher ASA score. In those situations, blood transfusion may be allowed by a routine judgement, as given by our computer algorithm. However, physician experts believed patients’ condition could be controlled with a cautious clinical management without a need for blood transfusion. Such a human judgement was built over years’ of clinic experiences and comprehension on the prognosis of those health problems. Even thought the computer has the capacity of taking in more patient data into its algorithm, it is difficult for it to take dynamic variables into the decision making process like human experts. Therefore, current algorithm is not fully ready for replacing human expert in making the judgement on blood transfusion.

Having said that, we would like to protect the value of employing computing technology for checking the quality of blood transfusion and its value for saving human workload without scarifying the patient safety.

Unlike physicians trained in western countries who follow the strict guideline for blood transfusion, physicians and surgeons in China are not compliant to the guideline and they practice differently among different health systems and different level of hospitals. There is a high chance of identifying unappropriated prescriptions for blood transfusion. Therefore, in every level of health authorities in China, a large amount of manpower was employed to check the quality of blood transfusion cases. Taking a mid-sized province, Zhejiang with a 60-million population in China as an example, over 30 thousand of new blood transfusion cases were added to a provincial-wide database. A group of experts spend thousands of hours in reviewing the blood transfusion data, making it exceptionally challenging to monitor every single case.

When applying artificial intelligence to the blood management, we are glad to see that our algorithm worked well for predicting the appropriateness of blood transfusions; simply, more than 3600 cases of appropriate blood transfusions give the computer a sufficient chance to learn from a human with a high matching rate (99%). However, we still have a room to improve on those inappropriate cases (90.9%). It looks to us the size of the dataset is an important factor for educating a computer to act like a human.

Another factor that may contribute to the discrepancy between human and computer judgement may come from the data quality. Insufficient variables in some of the data entries or mistakes made when inputting the data to the computer may have caused disagreements between human and computer judgements. A more advanced and complex computer algorithm, such as a convolutional neural network, may be applied in the future in order to test if a better outcome can be achieved [17–19].

## Conclusion

The reported algorithm in this project allow computers to inspect blood transfusion cases in a speedy fashion. This new technology will help us to identify appropriate cases for blood transfusion with accuracy but is still accompanied with a level of false rate. Further research with a large sample size and improved data quality will be needed for upgrading our algorithm before using it for assisting physicians through a systematic approach. We expect this new approach will save hundreds of hours for physicians from tedious works and give them opportunity to service patients in a better way.

## Supplementary information

**Additional file 1: Appendix S1.** Details of the computer script for screening the appropriateness of blood transfusions.

## Data Availability

The datasets generated and analysed during the current study are not publicly available as ethics approval did not support public opening on the collected data but are available from the corresponding author on reasonable request.

## Abbreviations

WHO: the World Health Organization
Hb: hemoglobin
ASA: the American Society of Anesthesiologists
MLPNN: multilayer perceptron neural network
