# Supplementary material for: Computer algorithm can match physicians’ decisions about blood transfusions
Source: J Transl Med. 2019 Oct 10;17:340. doi: 10.1186/s12967-019-2085-y (PMC6785926; doi:10.1186/s12967-019-2085-y)
Supplement: Supplementary file 1 — Additional file 1: Appendix S1. Details of the computer script for screening the appropriateness of blood transfusions. [file 12967_2019_2085_MOESM1_ESM.docx]

Appendix: Details of the computer script.

import numpy as np

from numpy import array

import csv

import matplotlib as mpl

from mpl_toolkits.mplot3d import Axes3D

import matplotlib.pyplot as plt

from keras.datasets import imdb

from keras.models import Sequential

from keras.layers import Dense

from keras.layers import LSTM

from keras.layers import GRU

from keras.layers import Dropout

from keras.layers.embeddings import Embedding

from keras.preprocessing import sequence

from sklearn.metrics import classification_report,confusion_matrix

dataset_train= numpy.loadtxt("PBM-data_zheng0827_train.csv", delimiter=",")

xtrain = dataset_train [:,0:12]

ytrain = dataset_train [:,13:14]

dataset_test= numpy.loadtxt("PBM-data_zheng0827_test.csv", delimiter=",")

xtest = dataset_test[:,0:12]

ytest = dataset_test[:,13:14]

# create model

model = Sequential()

model.add(Dense(50, input_dim=13, activation='relu'))

model.add(Dense(2, activation='sigmoid'))

# Compile model

model.compile(loss='binary_crossentropy', optimizer='adam', metrics=['accuracy'])

print(model.summary())

# Fit the model

history=model.fit(xtrain, ytrain, epochs=50, batch_size=100, validation_data=(xtest, ytest))

# evaluate the model

score, acc = model.evaluate(xtest, ytest, batch_size=100)

print('Test score:', score)

print('Test accuracy:', acc)

scores = model.evaluate(xtest, ytest, verbose=0)

print("Accuracy: %.2f%%" % (scores[1]*100))

Y_pred = model.predict(xtest)

y_pred = np.argmax(Y_pred, axis=1)

incorrects = np.nonzero(model.predict_classes(xtest).reshape((-1,)) !=np.argmax(ytest, axis=1))

print(incorrects[0])

#Confussion Matrix

p=model.predict_proba(xtest) # to predict probability

target_names = ['class 0(Need)', 'class 1(No Need)']

print(classification_report(np.argmax(ytest,axis=1), y_pred,target_names=target_names))

print(confusion_matrix(
